# Evaluating the effect of database inflation in proteogenomic search on sensitive and reliable peptide identification

**DOI:** 10.1186/s12864-016-3327-5

**Published:** 2016-12-22

**Authors:** Honglan Li, Yoon Sung Joh, Hyunwoo Kim, Eunok Paek, Sang-Won Lee, Kyu-Baek Hwang

**Affiliations:** 1 School of Computer Science and Engineering, Soongsil University, Seoul, 06978 Republic of Korea; 2 Department of Computer Science, Hanyang University, Seoul, 04763 Republic of Korea; 3 Scientific Data Research Center, Korea Institute of Science and Technology Information, Daejeon, 34141 Republic of Korea; 4 Department of Chemistry, Research Institute for Natural Sciences, Korea University, Seoul, 02841 Republic of Korea

**Keywords:** False discovery rate, Proteogenomic search, Separate false discovery rate analysis, Simulation, Target-decoy approach, Model-based approach

## Abstract

**Background:**

Proteogenomics is a promising approach for various tasks ranging from gene annotation to cancer research. Databases for proteogenomic searches are often constructed by adding peptide sequences inferred from genomic or transcriptomic evidence to reference protein sequences. Such inflation of databases has potential of identifying novel peptides. However, it also raises concerns on sensitive and reliable peptide identification. Spurious peptides included in target databases may result in underestimated false discovery rate (FDR). On the other hand, inflation of decoy databases could decrease the sensitivity of peptide identification due to the increased number of high-scoring random hits. Although several studies have addressed these issues, widely applicable guidelines for sensitive and reliable proteogenomic search have hardly been available.

**Results:**

To systematically evaluate the effect of database inflation in proteogenomic searches, we constructed a variety of real and simulated proteogenomic databases for yeast and human tandem mass spectrometry (MS/MS) data, respectively. Against these databases, we tested two popular database search tools with various approaches to search result validation: the target-decoy search strategy (with and without a refined scoring-metric) and a mixture model-based method. The effect of separate filtering of known and novel peptides was also examined. The results from real and simulated proteogenomic searches confirmed that separate filtering increases the sensitivity and reliability in proteogenomic search. However, no one method consistently identified the largest (or the smallest) number of novel peptides from real proteogenomic searches.

**Conclusions:**

We propose to use a set of search result validation methods with separate filtering, for sensitive and reliable identification of peptides in proteogenomic search.

**Electronic supplementary material:**

The online version of this article (doi:10.1186/s12864-016-3327-5) contains supplementary material, which is available to authorized users.

## Background

Proteogenomic search [1], i.e., searching tandem mass spectrometry (MS/MS) spectra against an integrated database consisting of reference proteins as well as protein sequences derived from genomic or transcriptomic evidence or hypotheses, is useful for identifying novel or sample-specific peptides. Typical approaches to the construction of proteogenomic databases include 6-frame translation of genome [2, 3] and extracting splicing information from RNA sequencing (RNA-seq) data [4–7]. In 6-frame translation of genome, peptide sequences are generated using each of the six possible frames. From the extracted splicing information, novel splice-junction peptide sequences could be obtained. These genomic or transcriptomic information sources are essential for identifying novel peptides, of which sequences are not contained in reference protein databases such as RefSeq [8] and UniProtKB [9]. Thus, proteogenomic search has been applied to various tasks such as discovering novel protein-coding regions [2, 10, 11], validation of gene annotation [12–15], and studying disease mechanisms for personalized diagnosis and treatments [16–18].

However, there are a number of challenges for proteogenomic search. Proteogenomic databases can be an order of magnitude larger than reference protein databases. For example, Woo and colleagues [6] constructed a 6-frame translation (102 MB) and a splice graph (410 MB) databases for *Caenorhabditis elegans*, which were respectively 7 and 28 times of a *C. elegans* reference protein database from UniProtKB. The increased size of proteogenomic databases demands a larger amount of computational resources, resulting in longer analysis time compared to the conventional proteomic database search.

Moreover, such inflation of proteogenomic databases makes it hard to apply widely used methods for controlling false discovery rates (FDRs) in peptide identification. In proteomic database search, the FDR of a search result is usually estimated by the target-decoy approach [19–21], in which a decoy database–consisting of reversed or shuffled version of the target protein database–is used. An inflated target database for proteogenomic search contains a large number of spurious peptide sequences. For example, most of the peptide sequences obtained from 6-frame translation of a genome are not likely to be produced in vivo or in vitro. In this regard, the FDR in proteogenomic search is prone to underestimation, because random hits to the spurious peptide sequences are considered as target hits and their numbers are not negligible when the database inflation is significant. Furthermore, the size of decoy databases for proteogenomic search can be significantly larger than the size of decoy databases for conventional proteomic database search. An inflated decoy database could decrease the sensitivity of peptide identification at the same FDR, because the number of high-scoring decoy hits increases as the size of decoy database increases.

Since the early stage of proteogenomics, it has been well noted that proteogenomic searches would produce more erroneous identifications than proteomic database searches due to their database sizes [1, 22, 23]. Blakeley and colleagues [24] showed that database choice is an influencing factor on FDR estimation. They proposed to limit database size for an improved FDR estimation in the target-decoy approach. Krug and colleagues [25] showed that FDRs in proteogenomic search could be substantially underestimated, by using a 6-frame translated *Escherichia coli* genome. However, reliable and sensitive peptide identification methods applicable to various proteogenomic databases for organisms with still-evolving genomic information are still not available.

To systematically evaluate the effect of database inflation in proteogenomic search on peptide identification, we generated a set of simulated and real proteogenomic databases. Proteogenomic databases of varying sizes were simulated by adding decoy peptide sequences to reference protein databases. As real proteogenomic databases, 6-frame translated versions of the yeast and the human reference genomes and a splice graph database, constructed from a human RNA-seq data set, were used. A set of yeast and human MS/MS spectra were respectively searched against the simulated and real proteogenomic databases using two widely-used database search tools, i.e., X!Tandem [26] and Comet [27]. To validate the search results, the target-decoy search strategy [19, 21] and a mixture model-based method [28] were used and compared. The target-decoy search strategy was also tested with a refined scoring-metric calculated by the self-boosted Percolator [29]. The mixture model-based method assumes a mixture of score distributions for correct and incorrect peptide identifications. On the contrary, the other methods require minimal distributional assumptions on peptide-spectrum match (PSM) scores. Additionally, we examined the effect of separate filtering of known and novel peptides with each of these methods. The separate filtering method has been suggested for proteogenomic search, considering the difference in the probability of identifying known and novel peptides [1]. Our evaluation and comparison results of various peptide identification approaches applied to various proteogenomic databases provide insight into peptide identifications in proteogenomics.

## Methods

### MS/MS data set

We used a yeast MS/MS data set generated and studied by Joo and colleagues [30]. Briefly, the data set was obtained from a yeast cell lysate, which was digested by trypsin and then separated by MudPIT [31]. For the MS/MS analysis, an LTQ-Orbitrap hybrid mass spectrometer was used. The yeast data set contained 63,031 MS/MS scans. We also used a human MS/MS data set generated from a human gastric tissue sample. The tissue sample was obtained from a Korean gastric cancer patient, who signed internal review board (IRB)-approved informed consents. The human sample was digested by trypsin and analyzed using a quadrupole orbitrap mass spectrometer (Q Exactive, Thermo Scientific, Bremen, Germany) coupled with a dual online ultrahigh pressure liquid chromatography system (see Additional file 1: Supplementary methods for details on sample preparation and liquid chromatography (LC)-MS/MS experiments). The resulting data set contained 139,629 MS/MS spectra.

### Databases consisting of reference protein sequences

A yeast “target” protein database (1T_y_), including 179 common contaminants and 6619 *S. cerevisiae* proteins downloaded from Swiss-Prot (07/2012), was constructed. The total length of protein sequences in 1T_y_ was 3,062,279 amino acid (AA). A human target protein database (1T_h_), containing UniProt human protein sequences (05/2013; 90,191 entries) and 179 common contaminants, was built. The total length of protein sequences in 1T_h_ was 35,856,033 AA. For simulated proteogenomic database construction and FDR estimation (see Database construction for simulated proteogenomic search and Database search and validation of search result), we used decoy databases 1D_y_ and 1D_h_, which were constructed by ‘pseudo-reversing’ [19] or ‘pseudo-shuffling’ the protein sequences in 1T_y_ and 1T_h_, respectively. To construct the decoy databases, all the fully-tryptic peptides (with maximum missed cleavage value of two) from the target protein databases were extracted. Then, each of the extracted peptides was reversed (pseudo-reversing) or randomly permuted (pseudo-shuffling), preserving the length and the amino acid composition of the original peptide. By modeling the null hypothesis (i.e., incorrect PSM), decoy databases can be used for *p*-value calculation and FDR estimation in peptide identification [32].

### Database construction for real proteogenomic search

Two types of real proteogenomic target databases were used in the experiments: 6-frame translation databases for yeast (6FTT_y_) and human (6FTT_h_) as well as a splice graph database for human (SGT_h_). 6FTT_y_ was constructed by 6-frame translation of the yeast whole-genome sequences (04/2014) downloaded from http://downloads.yeastgenome.org/sequence/S288C_reference/chromosomes/fasta/. 6FTT_h_ was generated based on 6-frame translation of the human reference genome (hg19) downloaded from ftp://ftp.ensembl.org/pub/release-71/fasta/homo_sapiens/dna/. Both 6FTT_y_ and 6FTT_h_ were constructed using Cancer Proteogenomics Tools developed by Woo and colleagues [6] (downloaded from http://proteomics.ucsd.edu/software-tools/splicedb-splice-graph-proteomics-tools/). It translates regions in genome, between start and stop codons, and ignores any splicing events. The length of proteins generated by the tool is usually shorter than the length of reference proteins. In total, 6FTT_y_ contained 114,386 proteins, corresponding to 2,010,708 fully-tryptic unique peptides (with minimum length of eight AA and maximum missed cleavage value of 2). Among the 688,452 fully-tryptic peptides in 1T_y_, 677,777 (98.4%) existed in 6FTT_y_. The number of proteins in 6FTT_h_ was 34,041,059, corresponding to 389,586,415 fully-tryptic unique peptides (with minimum length of eight AA and maximum missed cleavage value of 2). Among the 3,118,351 fully-tryptic peptides in 1T_h_, 1,851,052 (59.4%) were contained in 6FTT_h_.

SGT_h_ was constructed using an RNA-seq data set obtained from the same tissue sample, used for generating the human MS/MS data set (see MS/MS data set). The RNA-seq data set (binary sequence alignment/map (BAM) file) contained 41,353,547 reads mapped onto the human reference genome (hg19). A splice graph, of which nodes and edges respectively denote exons and splice junctions, was built using the read mapping information in the RNA-seq data set. From the splice graph, protein sequences (i.e., splice graph targets) for database search were extracted. We used Cancer Proteogenomics Tools for constructing SGT_h_ as in the studies by Woo and colleagues [6] (see Additional file 1: Supplementary methods for details on the RNA-seq analysis and splice graph database construction). SGT_h_ included 264,426 splice graph targets and 90,370 entries from 1T_h_. Decoy databases for the three target proteogenomic databases were created by pseudo-reversing (see Databases consisting of reference protein sequences) and are denoted as 6FTD_y_, 6FTD_h_, and SGD_h_, respectively.

### Database construction for simulated proteogenomic search

We assumed that the majority of the newly added peptide sequences to 6-frame translation and splice graph databases, apart from reference protein sequences, are not real target sequences but random sequences. For example, the proportion of novel peptides identified from a recent proteogenomic search [7] was 0.8% of the total peptides identified, although the size of proteogenomic database was more than 60 times larger than that of the reference protein database. To test our hypothesis, we constructed simulated proteogenomic databases containing varying numbers of “simulated novel” proteins, which were generated by the decoy database generation methods (see Databases consisting of reference protein sequences).

A simulated proteogenomic target database for yeast, 1T*n*D_y_, was constructed by combining 1T_y_ and a decoy database, *n*D_y_, of which size is *n* times larger than the size of 1T_y_. To build *n*D_y_, one pseudo-reversed version of 1T_y_, and (*n* – 1) pseudo-shuffled versions of 1T_y_ were generated and merged. For example, 1T5D_y_ consisted of 1T_y_, 1D_y_ (pseudo-reversed), and 4D_y_ (pseudo-shuffled). When simulating proteogenomic search with target-decoy approaches, the decoy database for 1T*n*D_y_ was constructed by combining (*n* + 1) pseudo-shuffled versions of 1T_y_, and was denoted as (*n* + 1)D_y_. Then, a search was performed against ‘1T*n*D_y_ + (*n* + 1)D_y_’. For example, the yeast MS/MS data set was searched against ‘1T5D_y_ + 6D_y_’, where 1T5D_y_ and 6D_y_ respectively correspond to a simulated target proteogenomic database and a same-sized decoy database. The same procedure was used for simulating proteogenomic search for human. Additional file 2: Figure S1 illustrates the entire workflow of simulated proteogenomic database construction. In the experiments, we used 1, 2, and 5 for the values of *n* to evaluate the effect of database inflation. Tables 1 and 2 summarize the size of the databases containing reference protein sequences as well as the real and simulated proteogenomic databases used in our experiments.

**Table 1:** Size of proteomic, simulated proteogenomic, and real proteogenomic databases for yeast

Database (target + decoy)		# Target (AA)	# Decoy (AA)
Proteomic	1T_y_ + 1D_y_	3,062,279	3,062,279
Simulated proteogenomic	1T1D_y_ + 2D_y_	6,124,558	6,124,558
	1T2D_y_ + 3D_y_	9,186,837	9,186,837
	1T5D_y_ + 6D_y_	18,373,674	18,373,674
Real proteogenomic	6FTT_y_ + 6FTD_y_	9,654,965	9,654,965

Database sizes are measured by total length (AA) of contained peptides. 1T_y_: yeast reference protein database. *n*D_y_: decoy database of which size is *n* times of 1T_y_. 6FTT_y_: proteogenomic database constructed by 6-frame translation of yeast genome. 6FTD_y_: decoy database for 6FTT_y_

**Table 2:** Size of proteomic, simulated proteogenomic, and real proteogenomic databases for human

Database (target + decoy)		# Target (AA)	# Decoy (AA)
Proteomic	1T_h_ + 1D_h_	35,856,033	35,856,033
Simulated proteogenomic	1T1D_h_ + 2D_h_	71,712,066	71,712,066
	1T2D_h_ + 3D_h_	107,568,099	107,568,099
	1T5D_h_ + 6D_h_	215,136,198	215,136,198
Real proteogenomic	6FTT_h_ + 6FTD_h_	2,136,069,837	2,136,069,837
	SGT_h_ + SGD_h_	123,364,545	123,364,545

Database sizes are measured by total length (AA) of contained peptides. 1T_h_: human reference protein database. *n*D_h_: decoy database of which size is *n* times of 1T_h_. 6FTT_h_: proteogenomic database constructed by 6-frame translation of human genome. 6FTD_h_: decoy database for 6FTT_h_. SGT_h_: proteogenomic database constructed by splicing information from human RNA sequencing data. SGD_h_: decoy database for SGT_h_

The pseudo-shuffling method could introduce an extra level of redundancy into decoy databases by producing multiple peptides of a same sequence. This can be especially problematic in simulated proteogenomic search, where a large-sized decoy database is generated by pseudo-shuffling. Thus, we checked the level of redundancy in the decoy databases used in our experiments for simulated proteogenomic search. The proportion of redundant peptides in the generated decoy databases was less than 0.74 and 1.50% for yeast and human, respectively (see Additional file 3: Table S1). These numbers are still smaller than the proportion of redundant peptides in the reference protein databases, 1T_y_ and 1T_h_: 2.24 and 59.62%, respectively.

### Database search and validation of search result

Two database search tools, X!Tandem [26] and Comet [27], were used in the experiments. For the yeast MS/MS data set, 3 Da peptide mass tolerance, 1 Da MS/MS mass tolerance, and semi-tryptic option, were assigned to the two search tools. The human MS/MS data set was also searched by X!Tandem and Comet with 15 ppm parent mass tolerance, 0.03 Da fragment mass tolerance, and fully-tryptic option. For both data sets, one fixed modification at Cys (Carbamidomethyl, +57.02146) and one variable modification at Met (Oxidation, +15.99492) were allowed.

We applied the target-decoy search strategy (TD) to search result validation. Only the PSMs with minimum peptide length of eight AA were validated based on the PSM score: E-value for X!Tandem and XCorr for Comet. The FDR was estimated by *N*_*D*_/*N*_*T*_, where *N*_*D*_ and *N*_*T*_ respectively denote the number of decoy hits and the number of target hits above the score threshold. Furthermore, the effect of scores used in TD was tested by the self-boosted Percolator (BP) [29]. BP is an improved version of Percolator [33], in which the sensitiveness of Percolator to initial PSM ranking decreases by repeatedly applying the semi-supervised learning procedure with different labeling of training examples. We also examined a mixture model-based method (MB) for identifying high-confidence peptides. PeptideProphet [28] (trans-proteomic pipeline (TPP) version 4.7.1) in semi-parametric mode (with minimum peptide length of eight AA, minimum peptide probability of 0, and the accurate mass binning option) was applied to FDR estimation. As recommended, the Gumbel distribution and the Gaussian distribution were chosen for modeling the discriminant function values for incorrect PSMs in X!Tandem and Comet, respectively. After a mixture model was fitted to a database search result, the FDR was estimated as follows [34]:

1 $$ \mathrm{F}\mathrm{D}\mathrm{R}=\frac{{\displaystyle {\sum}_{S_i\ge t}PE{P}_i}}{\left\{\#{S}_i:{S}_i\ge t\right\}}\kern0.5em , $$

where *S*_*i*_ and *PEP*_*i*_ respectively denote the discriminant function value and the posterior error probability of the *i* ^th^ PSM, and the denominator means the number of PSMs whose discriminant function values are equal to or larger than the cutoff value *t*. The discriminant function calculates a summarized quality-score for PSMs based on multiple features including search score, and is optimized for each search engine [34]. The posterior error probability of the *i* ^th^ PSM—the probability that it is incorrect given its discriminant function value *S*_*i*_ —was computed from the learned mixture model.

The three methods, TD, BP, and MB, were also applied to separate filtering of known and novel (or simulated novel) peptides (SepTD, SepBP, and SepMB; see Additional file 4: Figure S2). In SepTD, PSMs were divided into two groups—known and novel (or simulated novel)—after database search. Then, each PSM group was separately filtered by TD. For the semi-supervised machine learning in SepBP or SepMB, the PSMs of both reference (known) and non-reference (novel or simulated novel) protein sequences were used together as in BP or MB, because it was not possible to train a separate support vector machine or fit a separate mixture model using only the novel (or simulated novel) PSMs. After the machine learning step, PSMs were divided into known and novel (or simulated novel) groups. Then, each group was separately filtered as follows. In SepBP, the PSMs of each group were separately sorted by the recalibrated score from BP, and filtered by estimating the FDR as *N*_*D*_/*N*_*T*_. In SepMB, the PSMs of each group were separately sorted by (1 – posterior error probability), and filtered by estimating the FDR as *N*_*D*_/*N*_*T*_. In the experiments, 1% FDR cut-off was used for high-confidence peptide identification. We calculated peptide-level FDRs by considering only the highest-scoring PSM per peptide.

## Results and Discussion

### Comparison between simulated and real proteogenomic search results

To test the effectiveness of simulation experiments, peptide identification results were compared between the following simulated and real proteogenomic database pairs of similar sizes: ‘1T2D_y_ + 3D_y_’ (9,186,837 + 9,186,837 AA) and ‘6FTT_y_ + 6FTD_y_’ (9,654,965 + 9,654,965 AA) for yeast, and ‘1T2D_h_ + 3D_h_’ (107,568,099 + 107,568,099 AA) and ‘SGT_h_ + SGD_h_’ (123,364,545 + 123,364,545 AA) for human (see Tables 1 and 2).

**Fig. 1:**
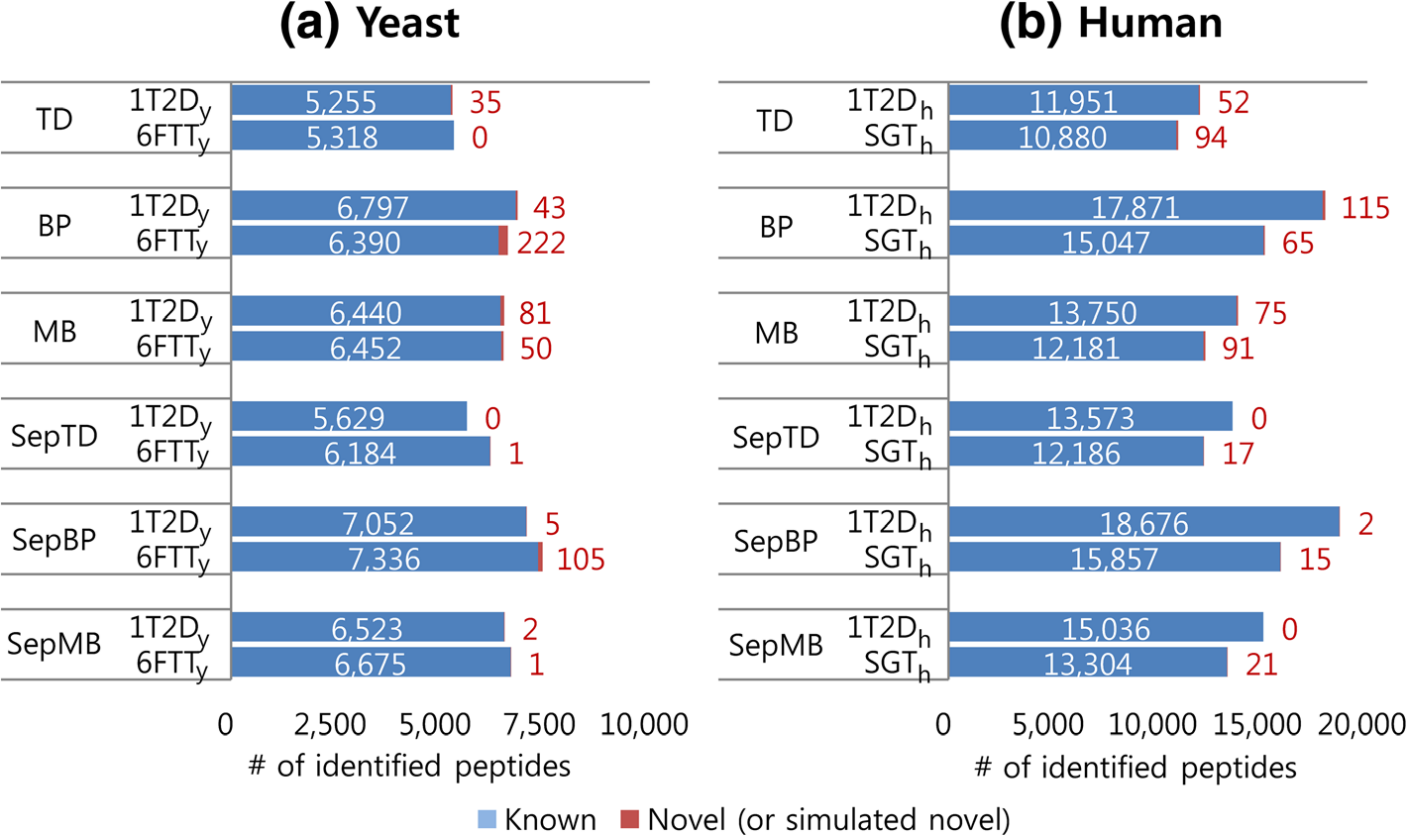
Comparison of peptide identification results between a pair of simulated and real proteogenomic databases of similar sizes for yeast (1T2D_y_ and 6FTT_y_) (**a**) and human (1T2D_h_ and SGT_h_) (**b**). Database searches were performed using X!Tandem. The number of peptides at 1% FDR is shown. TD: target-decoy search strategy. BP: TD with a refined scoring-metric calculated by the self-boosted Percolator. MB: mixture model-based method. SepTD, SepBP, and SepMB denote separate filtering of known and novel (or simulated novel) peptides with TD, BP, and MB, respectively. The *blue bars* and *numbers in white* denote the number of known peptides. The *red bars* and *numbers in red* denote the number of novel (or simulated novel) peptides

First, we examined the proportion of peptides from reference protein sequences (i.e., known peptides) among the peptide identification results, because we hypothesized that a substantial amount of peptides added to reference protein sequences for proteogenomic search would not be real target but random sequences (see Database construction for simulated proteogenomic search). Figure 1 shows the number of known and novel (or simulated novel) peptides at 1% FDR identified from the search results using X!Tandem. As expected, most peptides from the simulated and the real proteogenomic searches were known peptides: more than 98.76% for ‘1T2D_y_ + 3D_y_’, 96.64% for ‘6FTT_y_ + 6FTD_y_’, 99.36% for ‘1T2D_h_ + 3D_h_’, and 99.14% for ‘SGT_h_ + SGD_h_’. The results using Comet were also similar (Additional file 5: Figure S3).

The total number of known and novel (or simulated novel) peptides identified from the simulated and real proteogenomic databases of similar sizes was also similar in most cases. For yeast, the difference was from 51 to 556 (corresponding to 0.95 to 8.99% of the peptides identified from ‘6FTT_y_ + 6FTD_y_’) when X!Tandem was used (Fig. 1(a)). The difference in the results for yeast obtained using Comet was also small (from 0.90 to 4.41%), except for the results validated by SepTD and SepBP, in which the difference was 32.63 and 15.51% of the number of peptides identified from ‘6FTT_y_ + 6FTD_y_’, respectively (Additional file 5: Figure S3(a)). In these two cases, the increase in the number of identified peptides by separate filtering of known and novel (or simulated novel) peptides was much larger for ‘6FTT_y_ + 6FTD_y_’ than for ‘1T2D_y_ + 3D_y_’. For the human data set, the difference in the number of identified peptides was 9.38 to 19.02% (for X!Tandem) and 2.80 to 13.86% (for Comet) of the number of peptides identified from ‘SGT_h_ + SGD_h_’ (Fig. 1(b) and Additional file 5: Figure S3(b)). It must be noted that the size difference between the simulated and real proteogenomic databases was 4.85% (‘1T2D_y_ + 3D_y_’ and ‘6FTT_y_ + 6FTD_y_’) and 12.80% (‘1T2D_h_ + 3D_h_’ and ‘SGT_h_ + SGD_h_’).

Thus, we observed that proteogenomic search against simulated and real proteogenomic databases of similar sizes produced similar results with regard to the proportion of known peptides identified from reference protein sequences as well as the total number of identified peptides at the same FDR in most cases. These results suggest that we could use simulated proteogenomic databases for quantitatively examining the effect of database inflation on the sensitivity and reliability of peptide identifications.

### Sensitivity and reliability in simulated proteogenomic search

**Fig. 2:**
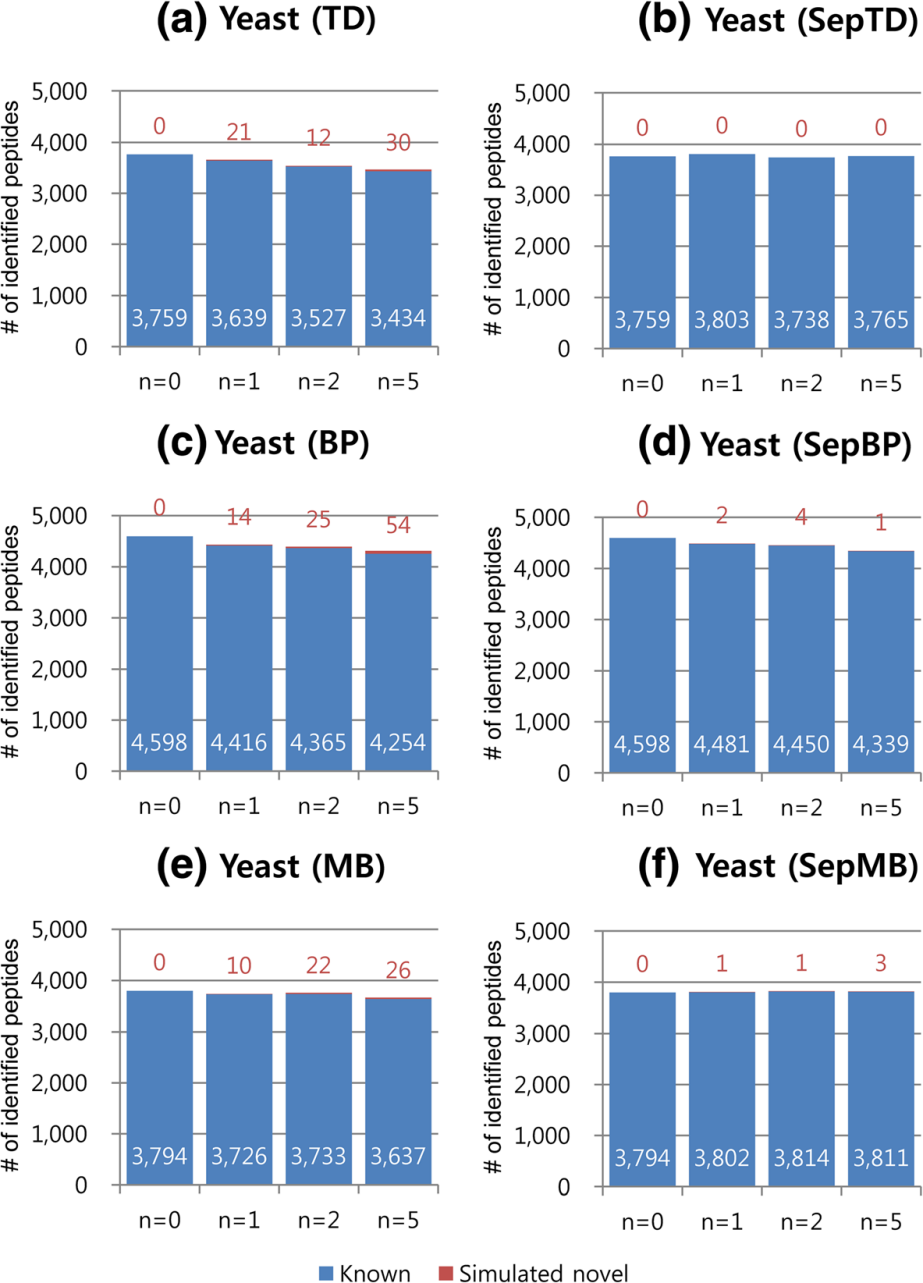
Peptide identification results from search against simulated proteogenomic databases for yeast (1T*n*D_y_) using X!Tandem (*n* = 0, 1, 2, and 5). The number of peptides with charge 2+ at 1% FDR is shown. TD: target-decoy search strategy (**a**). BP: TD with a refined scoring-metric calculated by the self-boosted Percolator (**c**). MB: mixture model-based method (**e**). SepTD (**b**), SepBP (**d**), and SepMB (**f**) denote separate filtering of known and simulated novel peptides with TD, BP, and MB, respectively. The *blue bars* and *numbers in white* denote the number of known peptides. The *red bars* and *numbers in red* denote the number of simulated novel peptides

**Fig. 3:**
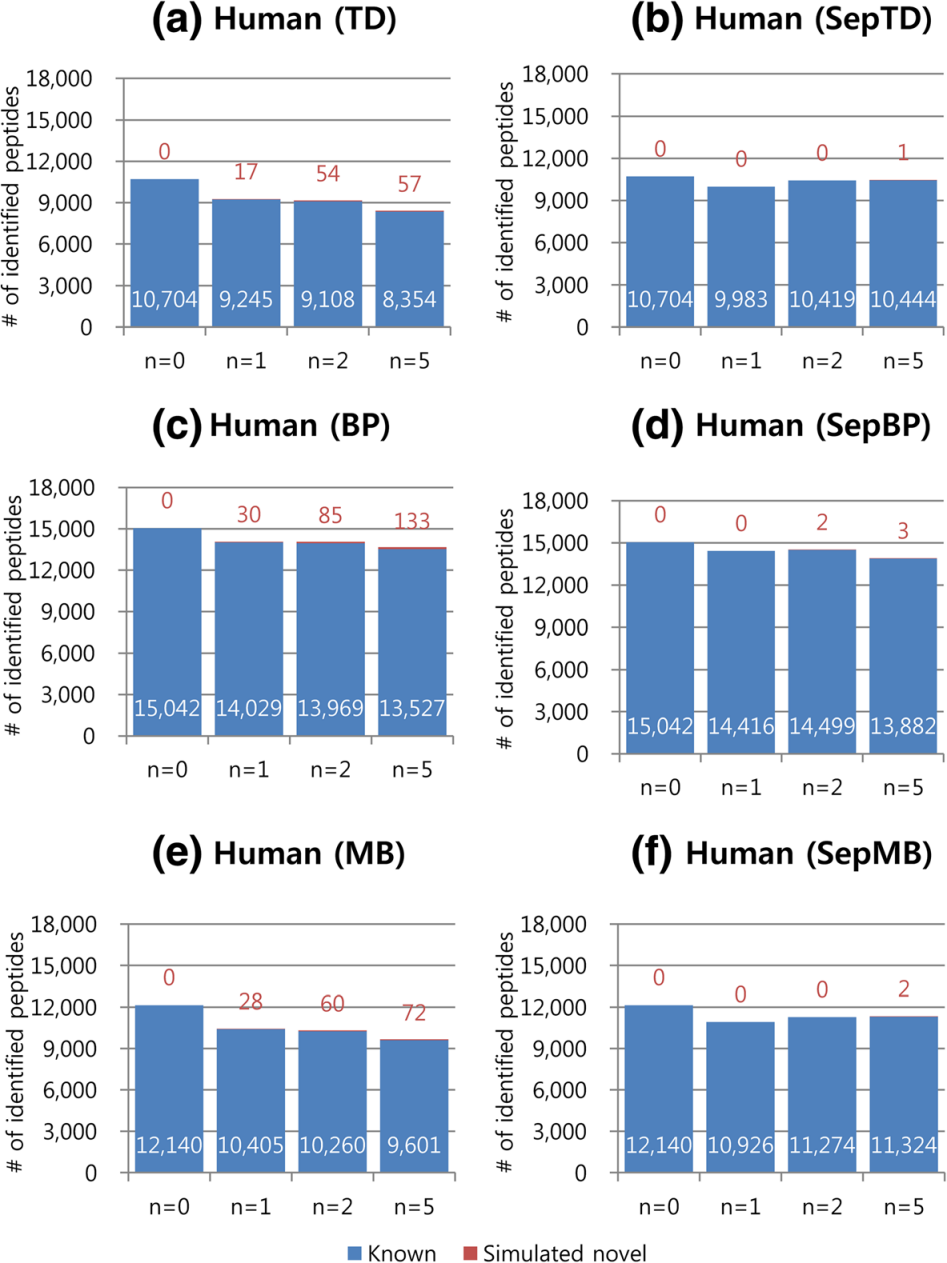
Peptide identification results from search against simulated proteogenomic databases for human (1T*n*D_h_) using X!Tandem (*n* = 0, 1, 2, and 5). The number of peptides with charge 2+ at 1% FDR is shown. TD: target-decoy search strategy (**a**). BP: TD with a refined scoring-metric calculated by the self-boosted Percolator (**c**). MB: mixture model-based method (**e**). SepTD (**b**), SepBP (**d**), and SepMB (**f**) denote separate filtering of known and simulated novel peptides with TD, BP, and MB, respectively. The *blue bars* and *numbers in white* denote the number of known peptides. The *red bars* and *numbers in red* denote the number of simulated novel peptides

We investigated the effect of database inflation in proteogenomic search on sensitivity and reliability of peptide identification by using simulated target-protein databases for proteogenomic search, comprised of reference (1T_y_ or 1T_h_) and simulated novel (*n*D_y_ or *n*D_h_) protein sequences. Here, sensitivity denotes the number of identified peptides from target protein databases. As decoy databases for simulated proteogenomic search, (*n* + 1)D_y_ or (*n* + 1)D_h_ was used (see Database construction for simulated proteogenomic search). Figures 2 and 3 show the peptide identification results (charge 2+ and FDR 1%) using X!Tandem from simulated proteogenomic databases of varying sizes (*n* = 0, 1, 2, and 5) for yeast and human, respectively. Overall, the number of peptides identified by using TD, BP, and MB decreased as the number of added decoy-peptides to the target database increased in most cases. For example, the number of peptides identified by search against ‘1T*n*D_y_ + (*n* + 1)D_y_’ (FDR 1% controlled by TD) decreased from 3759 to 3434 as *n* increased from 0 to 5 (Fig. 2(a)). When *n* equals 5, the decrease rate in the number of identified peptides was 8.65% (TD for yeast), 7.48% (BP for yeast), 4.14% (MB for yeast), 21.95% (TD for human), 10.07% (BP for human), and 20.91% (MB for human) (Figs. 2(a), (c), (e), 3(a), (c), and (e)). Thus, it seems that the inflated database could substantially deteriorate the sensitivity of proteogenomic search when known and simulated novel peptides are filtered together. However, the effect of database inflation was substantially attenuated by separate filtering of known and simulated novel peptides. The number of peptides identified by using SepTD or SepMB did not decrease while the database size increased (Figs. 2(b), (f), 3(b), and (f)). The decrease rate in the number of peptides identified using SepBP was smaller than the decrease rate in the results using BP (Figs. 2(c), (d), 3(c), and (d)). From the results on 3+ peptides (Additional file 6: Figure S4 and Additional file 7: Figure S5) and the results obtained using Comet (Additional file 8: Figure S6, Additional file 9: Figure S7, Additional file 10: Figure S8, and Additional file 11: Figure S9), we observed similar tendencies. Therefore, it is essential to filter known and novel peptides separately for high sensitivities in proteogenomic search.

Among the three separate filtering methods, SepBP identified the largest number of peptides from the search results using X!Tandem (Figs. 2 and 3; Additional file 6: Figure S4 and Additional file 7: Figure S5). In most cases, SepBP and SepMB identified larger numbers of peptides than SepTD from the search results using Comet (Additional file 8: Figure S6, Additional file 9: Figure S7, Additional file 10: Figure S8, and Additional file 11: Figure S9). Thus, machine learning-based methods for search result validation seem to improve the sensitivity in proteogenomic search.

We counted the number of simulated novel peptides identified from *n*D_y_ or *n*D_h_, because they are highly probable to be false positives. In most cases, the number of identified simulated-novel peptides increased as *n* increased (Figs. 2 and 3; Additional file 6: Figure S4, Additional file 7: Figure S5, Additional file 8: Figure S6, Additional file 9: Figure S7, Additional file 10: Figure S8, and Additional file 11: Figure S9). Thus, the database inflation in proteogenomic search could also deteriorate the reliability in peptide identification regardless of search result validation methods. However, the number of simulated novel peptides identified by TD, BP, or MB was always much larger than SepTD, SepBP, or SepMB, suggesting that separate filtering of known and novel peptides is also essential for improving the reliability in proteogenomic search. Among the three separate filtering methods, SepBP identified the largest number of simulated novel peptides in most cases. SepTD was the most conservative for simulated novel peptide identification.

### Sensitivity and reliability in real proteogenomic search

We examined and compared the six methods for search result validation using the three real proteogenomic databases: ‘6FTT_y_ + 6FTD_y_’ for yeast, ‘6FTT_h_ + 6FTD_h_’ and ‘SGT_h_ + SGD_h_’ for human (see Database construction for real proteogenomic search). Tables 3 and 4 respectively show the numbers of peptides with charge 2+ and with charge 3+ at 1% FDR, identified from the real proteogenomic search using X!Tandem. We observed that separate filtering of known and novel peptides consistently increased the number of identified peptides as in the results from simulated proteogenomic search. Moreover, the number of identified known-peptides increased, but the number of identified novel-peptides decreased by separate filtering (SepTD, SepBP, or SepMB). For example, the numbers of known and novel peptides (charge 2+) identified by TD from the search against ‘6FTT_h_ + 6FTD_h_’ were 4115 and 62, respectively (Table 3). From the same search result, SepTD identified 53.97% more known-peptides (6336) and 82.26% less novel-peptides (11). On average, 26.23% more known- and 89.18% less novel-peptides were identified by the three separate filtering methods (Tables 3 and 4). Considering the fact that novel peptides are more probable to be false positives than known ones, SepTD, SepBP, and SepMB seem to increase the sensitivity in the identification of known peptides while increasing the reliability in the identification of novel peptides. The results from the real proteogenomic searches using Comet were also similar (Additional file 12: Table S2 and Additional file 13: Table S3).

**Table 3:** Number of peptides with charge 2+ at 1% FDR identified from search against real proteogenomic databases using X!Tandem

Database (target + decoy)		TD	BP	MB	SepTD	SepBP	SepMB
6FTT_y_ + 6FTD_y_	Total	3,626	4,281	3,807	3,942	4,515	3,870
	Known	3,603	4,106	3,781	3,942	4,443	3,870
	Novel	23	175	26	0	72	0
6FTT_h_ + 6FTD_h_	Total	4,177	5,620	4,813	6,347	6,018	6,188
	Known	4,115	5,316	4,765	6,336	5,950	6,180
	Novel	62	304	48	11	68	8
SGT_h_ + SGD_h_	Total	8,034	11,059	9,152	8,957	11,150	9,552
	Known	7,966	11,016	9,087	8,940	11,136	9,531
	Novel	68	43	65	17	14	21

6FTT_y_ (or 6FTT_h_): proteogenomic database constructed by 6-frame translation of yeast (or human) genome. 6FTD_y_ (or 6FTD_h_): decoy database for 6FTT_y_ (or 6FTT_h_). SGT_h_: proteogenomic database constructed by splicing information obtained from human RNA sequencing data. SGD_h_: decoy database for SGT_h_. TD: target-decoy strategy. BP: target-decoy strategy using a refined score calculated by the self-boosted Percolator. MB: mixture model-based method. SepTD, SepBP, and SepMB denote separate filtering of known and novel peptides using TD, BP, and MB, respectively

**Table 4:** Number of peptides with charge 3+ at 1% FDR identified from search against real proteogenomic databases using X!Tandem

Database (target + decoy)		TD	BP	MB	SepTD	SepBP	SepMB
6FTT_y_ + 6FTD_y_	Total	1,705	3,452	2,403	2,054	4,072	2,490
	Known	1,697	3,407	2,385	2,053	4,071	2,489
	Novel	8	45	18	1	1	1
6FTT_h_ + 6FTD_h_	Total	1,436	3,001	1,022	2,363	3,055	2,356
	Known	1,413	2,959	1,005	2,348	3,044	2,352
	Novel	23	42	17	15	11	4
SGT_h_ + SGD_h_	Total	3,467	6,552	2,705	3,840	6,568	3,518
	Known	3,433	6,526	2,680	3,836	6,562	3,511
	Novel	34	26	25	4	6	7

6FTT_y_ (or 6FTT_h_): proteogenomic database constructed by 6-frame translation of yeast (or human) genome. 6FTD_y_ (or 6FTD_h_): decoy database for 6FTT_y_ (or 6FTT_h_). SGT_h_: proteogenomic database constructed by splicing information obtained from human RNA sequencing data. SGD_h_: decoy database for SGT_h_. TD: target-decoy strategy. BP: target-decoy strategy using a refined score calculated by the self-boosted Percolator. MB: mixture model-based method. SepTD, SepBP, and SepMB denote separate filtering of known and novel peptides using TD, BP, and MB, respectively

We compared the three separate filtering methods—SepTD, SepBP, and SepMB—regarding novel peptide identification. In Table 3, SepMB identified the smallest number of novel peptides with charge 2+ from the search against ‘6FTT_h_ + 6FTD_h_’ using X!Tandem. However, the same method identified the largest number of novel peptides with the same charge, from the search against ‘SGT_h_ + SGD_h_’ using the same database search tool. SepMB also identified the largest number of novel peptides (charge 2+) from the search against ‘6FTT_h_ + 6FTD_h_’ using Comet (Additional file 12: Table S2). Therefore, there does not seem to exist one specific method, which is the most (or the least) conservative for identifying novel peptides from real proteogenomic search, among the three filtering methods. In many cases, SepTD, SepBP, and SepMB identified similar numbers of novel peptides from the three real proteogenomic databases. However, SepBP identified much larger numbers (>50 more) of novel peptides with charge 2+ than SepTD and SepMB, from the searches against ‘6FTT_y_ + 6FTD_y_’ and ‘6FTT_h_ + 6FTD_h_’ using X!Tandem (Table 3). Thus, SepBP could produce different results compared with the other two methods in novel peptide identification from proteogenomic search.

**Fig. 4:**
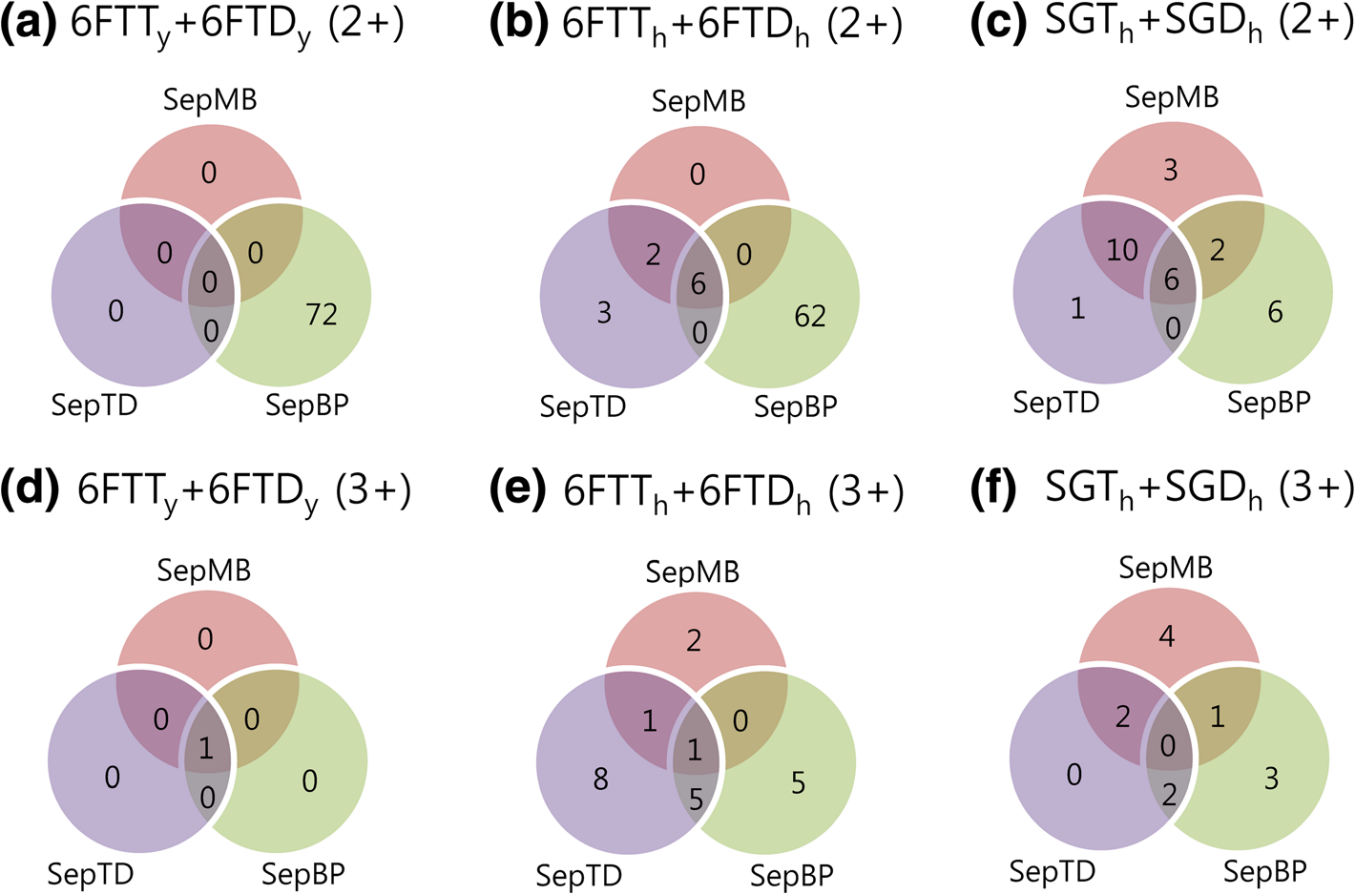
Comparison of novel peptides identified from real proteogenomic databases (‘6FTT_y_ + 6FTD_y_’ for yeast, ‘6FTT_h_ + 6FTD_h_’ and ‘SGT_h_ + SGD_h_’ for human). Database searches were performed by X!Tandem. The number of peptides with charge 2+ ((**a**), (**b**), and (**c**)) and 3+ ((**d**), (**e**), and (**f**)) at 1% FDR is shown. Three separate filtering methods (SepTD, SepBP, and SepMB) were used for search result validation

We also compared the novel peptides identified at 1% FDR by SepTD, SepBP, and SepMB. Figure 4 and Additional file 14: Figure S10 show the comparison results for X!Tandem and Comet, respectively. In most cases, the number of novel peptides commonly identified by the three filtering methods was small. The three methods commonly identified one novel-peptide (charge 3+) from the search against ‘6FTT_y_ + 6FTD_y_’ (see Fig. 4(d) and Additional file 14: Figure S10(d)). Except for this case, the proportion of commonly-identified novel peptides was less than 30%, suggesting that the sensitivity in novel peptide identification could be improved by combining results from multiple methods for separate filtering of known and novel peptides.

With regard to the identification of known peptides from proteogenomic search, SepBP identified larger numbers of peptides than SepTD and SepMB in most cases. From the search results obtained using Comet, SepBP identified 6.23 to 65.82% more known-peptides compared to SepTD or SepMB (Additional file 12: Table S2 and Additional file 13: Table S3). Only for the human data set searched against ‘6FTT_h_ + 6FTD_h_’ using X!Tandem, SepBP identified a smaller number (5950) of known peptides (charge 2+) compared with SepTD (6336) and SepMB (6180), respectively (Table 3). In many cases, SepTD and SepMB identified similar numbers of known peptides, except for the case of identifying known peptides (charge 3+) from the search against ‘6FTT_y_ + 6FTD_y_’ using X!Tandem, where SepMB identified 21.24% more peptides than SepTD (Table 4).

Besides the six search-result validation methods, we tested the two-stage FDR method [7] against ‘SGT_h_ + SGD_h_’. In the two-stage FDR method for proteogenomic search, only the spectra, not matched to reference protein sequences with a sufficient level of confidence, were searched against proteogenomic databases (see Additional file 1: Supplementary methods for more detailed description on the two-stage FDR method). Additional file 15: Table S4 compares the peptide identification results among TD, SepTD, and the two-stage FDR method. Similar to the separate filtering methods, the two-stage FDR method increased the number of identified known-peptides while decreasing the number of identified novel-peptides. SepTD and the two-stage FDR method identified similar numbers of novel peptides.

## Conclusions

Proteogenomic searches, originally suggested for gene annotation and validation, are now becoming a routine tool for many tasks including proteogenomic characterization of tumors. Since proteogenomic databases are inflated with a large number of spurious peptide sequences, it is important to accurately estimate the effect of such inflation on sensitive and reliable peptide identification. We evaluated the effect of database inflation in proteogenomic search using various simulated and real proteogenomic databases. Two popular database search tools with six approaches to search result validation were tested. First, we showed that the number of peptides identified from simulated and real proteogenomic databases of similar sizes is also similar, confirming the effectiveness of using simulated proteogenomic databases in estimating sensitivity and reliability of various search and validation strategies. Then, the relationship between the database size and the number of identified peptides was examined using simulated proteogenomic databases. When known and simulated-novel peptides were filtered together, the number of peptides at the same FDR decreased as the (target + decoy) database size increased. However, the results from separate filtering of known and simulated-novel peptides were almost not influenced by changes in database sizes. Moreover, the separate filtering methods effectively removed most of the simulated-novel peptides, which are highly likely to be false positives. Finally, the sensitivity and reliability of real proteogenomic search was examined using 6-frame translated versions of the yeast and the human genomes and a splice graph database constructed using human RNA-seq data. As in the results from simulated proteogenomic search, separate filtering of known and novel peptides increased the number of identified known-peptides while decreasing the number of identified novel-peptides, compared with the methods which filter known and novel peptides together. Therefore, separate filtering of known and novel peptides is strongly recommended for proteogenomic database search. Among the three separate filtering methods, SepBP generally identified the largest number of peptides, suggesting that semi-supervised machine learning could be effective in increasing the sensitivity of proteogenomic search. In terms of novel peptide identification, the three separate filtering methods usually identified similar numbers of novel peptides; however, no one method consistently identified the largest (or the smallest) number of novel peptides. Furthermore, the number of novel peptides commonly identified by the three methods was not large, suggesting that false negatives could be an issue even in novel peptide identification. In order to reduce the false negatives, one can apply multiple separate filtering methods to a proteogenomic search result and combine the novel peptides identified by each method. As a conclusion, we propose to use two or more methods for search result validation with separate filtering of known and novel peptides, for maximizing the sensitivity and reliability in proteogenomic search.

## Additional files

Additional file 1: Supplementary methods. Descriptions on the human MS/MS data set, RNA sequencing analysis, construction of splice graph database, and two-stage FDR method. (DOCX 20 kb)
Additional file 2: Figure S1. Workflow for generating a simulated proteogenomic database for yeast. (DOCX 743 kb)
Additional file 3: Table S1. Proportion of redundant peptides in decoy databases for simulated proteogenomic search. (DOCX 14 kb)
Additional file 4: Figure S2. Workflow of separate filtering FDR methods (DOCX 55 kb)
Additional file 5: Figure S3. Comparison of peptide identification results between a pair of simulated and real proteogenomic databases of similar sizes for yeast and human. (DOCX 61 kb)
Additional file 6: Figure S4. Peptide (charge 3+) identification results from search against simulated proteogenomic databases for yeast using X!Tandem. (DOCX 73 kb)
Additional file 7: Figure S5. Peptide (charge 3+) identification results from search against simulated proteogenomic databases for human using X!Tandem. (DOCX 74 kb)
Additional file 8: Figure S6. Peptide (charge 2+) identification results from search against simulated proteogenomic databases for yeast using Comet. (DOCX 76 kb)
Additional file 9: Figure S7. Peptide (charge 2+) identification results from search against simulated proteogenomic databases for human using Comet. (DOCX 76 kb)
Additional file 10: Figure S8. Peptide (charge 3+) identification results from search against simulated proteogenomic databases for yeast using Comet. (DOCX 72 kb)
Additional file 11: Figure S9. Peptide (charge 3+) identification results from search against simulated proteogenomic databases for human using Comet. (DOCX 75 kb)
Additional file 12: Table S2. Number of peptides with charge 2+ at 1% FDR identified from search against real proteogenomic databases using Comet. (DOCX 16 kb)
Additional file 13: Table S3. Number of peptides with charge 3+ at 1% FDR identified from search against real proteogenomic databases using Comet. (DOCX 16 kb)
Additional file 14: Figure S10. Comparison of novel peptides identified from real proteogenomic databases. (DOCX 68 kb)
Additional file 15: Table S4. Number of identified peptides at 1% FDR from the human splice graph database. (DOCX 15 kb)

## Abbreviations

(*n* + 1)D_h_: Simulated proteogenomic decoy database for human
(*n* + 1)D_y_: Simulated proteogenomic decoy database for yeast
1T_h_: “Target” reference protein sequences for human
1T*n*D_h_: Simulated proteogenomic target database for human
1T*n*D_y_: Simulated proteogenomic target database for yeast
1T_y_: “Target” reference protein sequences for yeast
6FTD_h_: Six-frame translation decoy database for human
6FTD_y_: Six-frame translation decoy database for yeast
6FTT_h_: Six-frame translation target database for human
6FTT_y_: Six-frame translation target database for yeast
AA: Amino acid
BAM: Binary sequence alignment/map
BP: Self-boosted Percolator
FDR: False discovery rate
IRB: Internal review board
LC: Liquid chromatography
MB: Mixture model-based method
MS/MS: Tandem mass spectrometry
PSM: Peptide-spectrum match
RNA-seq: RNA sequencing
SepBP: Separated filtering of known and novel peptides using self-boosted Percolator
SepMB: Separated filtering of known and novel peptides using mixture model-based method
SepTD: Separated filtering of known and novel peptides using target-decoy search strategy
SGD_h_: Splice graph decoy database for human
SGT_h_: Splice graph target database for human
TD: Target-decoy search strategy
TPP: Trans-proteomic pipeline
