# Supplementary material for: Evaluating the effect of database inflation in proteogenomic search on sensitive and reliable peptide identification
Source: BMC Genomics. 2016 Dec 22;17(Suppl 13):1031. doi: 10.1186/s12864-016-3327-5 (PMC5259817; doi:10.1186/s12864-016-3327-5)
Supplement: Additional file 1: — Supplementary methods. Descriptions on the human MS/MS data set, RNA sequencing analysis, construction of splice graph database, and two-stage FDR method. (DOCX 20 kb) [file 12864_2016_3327_MOESM1_ESM.docx]

**Supplementary methods**

**Human gastric cancer data set**

*Sample preparation* – A human gastric tissue was obtained from a Korean gastric cancer patient, who signed IRB-approved informed consents. The tissue was washed with 0.1 M Tris-HCl (pH 7.6) and was homogenized in 0.1 M Tris-HCl using a mini-beadbeater (Biospec Products, Bartlesville, OK). The supernatant was further lysed by sonication for 15 s using a probe sonicator (Qsonica, Newtown, CT) three times. The protein extract was transferred to a new tube and dissolved in an SDT buffer (4% sodium dodecyl sulfate in 0.1 M Tris-HCl pH 7.6 and 0.1 M dithiothreitol). A slightly modified filter-aided sample preparation (FASP) method [1] was used for digestion. Briefly the proteins were reduced at 37 °C for 2 h and boiled at 100 °C for 10 min. The resultant proteins were mixed with 200 μL of 8 M urea in 0.1 M Tris–HCl (pH 8.5) on a 30 K membrane filter (Microcon device, YM-30, Millipore, MA). The filters were centrifuged at 14,000 × g at 20 °C for 50–60 min. 100 μL of 50 mM iodoacetamide in 8 M urea was added for alkylation. The concentrate was washed with 200 μL of 8 M urea and subsequently washed with 100 μL of 50 mM ammonium bicarbonate by centrifugation. The protein concentrate was digested using a trypsin (Promega, Madison, WI, 1:50 enzyme to protein ratio) with gentle shaking in a Thermomixer (Comfort, Eppendorf, Hamburg, Germany) at 600 rpm for 1 min before overnight digestion without shaking at 37 °C. After the first digestion, the trypsin (1:100 of enzyme to protein ratio) was added for additional digestion. The resultant peptides were eluted from the filter and vacuum-dried using a SpeedVac concentrator (Centra-Vac VS-802, Vision, Korea) and the dried peptides were stored at −80 °C until LC–MS/MS experiments.

*LC-MS/MS experiments* – The tryptic peptides were analyzed using a quadrupole orbitrap mass spectrometer (Q Exactive, Thermo Scientific, Bremen, Germany) coupled with a dual online ultrahigh pressure liquid chromatography system, which was previously described [2]. A 300-min gradient (1% to 40 % solvent B for 270 min, 40% to 80 % for 10 min, 80% for 10 min and 1% for 10 min) was used at flow rate of 300 nL/min. Solvent A and B were 0.1% formic acid in water and 0.1% formic acid in acetonitrile. Analytical column (75 μm × 100 cm) and solid phase extraction column (150 μm × 3 cm) were fabricated in-house through slurry packing with C18-bonded particle (Jupiter, 3 μm, 300 Å, Phenomenex) [2]. The operating temperature of the analytical column was 60 °C. The eluted peptides were ionized through a home-built nano-electrospray source at an electric potential of 2.2 kV. Full MS scans were acquired at a resolution of 70,000 with a mass range of 300–1750 Th. A target value of automatic gain control (AGC) and maximum injection time (IT) were 1.0 × 10^6^ and 20 ms, respectively. Up to ten most abundant ions in full mass scan were isolated with an isolation window of 1.6 Th and fragmented using higher-energy collisional dissociation (HCD). A stepped normalized collision energy of 25 with 20% was used. Ions with +1 charge state were excluded and intensity threshold for fragmentation was 1.7 × 104. MS/MS scans were acquired at a resolution of 17,500. The AGC target value and maximum IT were 1.0 × 10^6^ and 60 ms, respectively. The resulting data set contained 139,629 MS/MS spectra.

**RNA sequencing analysis and construction of splice graph database for human sample**

We used mRNA sequencing results for constructing a splice graph database. The same gastric cancer tissue that was used for LC-MS/MS analyses as described in the previous section was processed to produce RNA sequencing (RNA-seq) results. Total RNA was isolated from the tumor sample using mirVana kit (Ambion, Carlsbad, MA). For mRNA sequencing, the library was prepared using 1 μg of DNase I-treated total RNA using Illumina TruSeq kit (Illumina Cat. No. 1502062, San Diego, CA), and the paired-end sequencing was performed using the Illumina HiSeq 2000. The resulting data set contained 41,353,850 paired-end reads (2x84 bp). These RNA-seq reads were mapped to a human reference genome GRCh37 (07/02/2014) with --read-realign-edit-dist 2 option and the default parameters by TopHat (version 2.0.11) [3]. In total, 41,353,547 reads were mapped. Then, a splice graph database was constructed using the mapped reads, and the results were converted to fasta files using the method proposed by Woo and colleagues [4] (downloaded from <http://proteomics.ucsd.edu/software-tools/splicedb-splice-graph-proteomics-tools/>). The detailed procedure is as follows. First, split (or spliced) reads were chosen from the read alignment file, based on CIGAR string. Then, multiple reads sharing the same intron boundary were merged into a single read. An initially empty splice graph, of which nodes and edges respectively correspond to exons and splice junctions, was constructed and updated read by read. The splicing information contained in each spliced read was used for updating the splice graph structure. Finally, a greedy approach was used for extracting protein sequences from the splice graph. Because most of the peptides in reference protein sequences (1T_h_) were not included in the splice graph database (less than 4%), 1T_h_ was added as in the study by Woo and colleagues [4].

**Two-stage FDR method against human splice graph database**

We used the two-stage FDR method [5] for peptide identification from the human splice graph database (SGT_h_). First, the human MS/MS data set was searched against the human reference protein database (1T_h_). Second, the spectra, identified at 1% PSM level FDR, were removed from the human MS/MS data set. Third, the remaining spectra were searched against the splice graph target sequences (‘SGT_h_ – 1T_h_’). Only the target-decoy search strategy was used for FDR estimation, because the number of identified novel-peptides was not enough for learning a support vector machine or fitting a mixture model. We reported the number of unique known-peptides at 1% PSM level FDR, and the number of novel peptides at 1% peptide level FDR.

**References**

[1] Wisniewski, J. R., Zougman, A., Nagaraj, N., Mann, M., Universal sample preparation method for proteome analysis. Nature methods 2009, 6, 359-362.

[2] Lee, H., Lee, J. H., Kim, H., Kim, S. J., et al., A fully automated dual-online multifunctional ultrahigh pressure liquid chromatography system for high-throughput proteomics analysis. Journal of chromatography. A 2014, 1329, 83-89.

[3] Trapnell, C., Pachter, L., Salzberg, S. L., TopHat: discovering splice junctions with RNA-Seq. Bioinformatics 2009, 25, 1105-1111.

[4] Woo, S., Cha, S. W., Merrihew, G., He, Y., et al., Proteogenomic database construction driven from large scale RNA-seq data. Journal of proteome research 2014, 13, 21-28.

[5] Woo, S., Cha, S. W., Na, S., Guest, C., et al., Proteogenomic strategies for identification of aberrant cancer peptides using large-scale next-generation sequencing data. Proteomics 2014, 14, 2719-2730.
