# Supplementary material for: Evaluating the effect of database inflation in proteogenomic search on sensitive and reliable peptide identification
Source: BMC Genomics. 2016 Dec 22;17(Suppl 13):1031. doi: 10.1186/s12864-016-3327-5 (PMC5259817; doi:10.1186/s12864-016-3327-5)
Supplement: Additional file 2: Figure S1. — Workflow for generating a simulated proteogenomic database for yeast. (DOCX 743 kb) [file 12864_2016_3327_MOESM2_ESM.docx]

Additional file 2: Figure S1


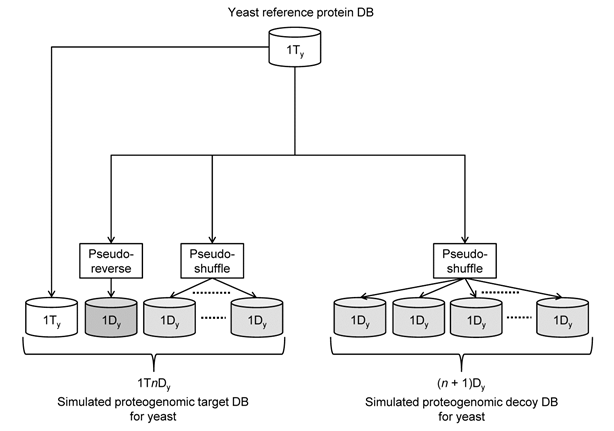


**Figure S1.** Workflow for generating a simulated proteogenomic database (target + decoy) for yeast, ‘1T*n*D_y_ + (*n* + 1)D_y_’. 1T_y_ denotes a target reference protein database for yeast, e.g., downloaded from Swiss-Prot. The target proteogenomic database, 1T*n*D_y_, is constructed by combining 1T_y_, 1D_y_ (pseudo-reversed), and (*n* – 1)D_y_ (pseudo-shuffled). The decoy proteogenomic database is constructed by combining (*n* + 1) pseudo-shuffled versions of 1T_y_, and is denoted as (*n* + 1)D_y_. Proteogenomic databases for human are constructed in the same manner, using a human reference protein database.
