# Supplementary material for: Evaluating the effect of database inflation in proteogenomic search on sensitive and reliable peptide identification
Source: BMC Genomics. 2016 Dec 22;17(Suppl 13):1031. doi: 10.1186/s12864-016-3327-5 (PMC5259817; doi:10.1186/s12864-016-3327-5)
Supplement: Additional file 3: Table S1. — Proportion of redundant peptides in decoy databases for simulated proteogenomic search. (DOCX 14 kb) [file 12864_2016_3327_MOESM3_ESM.docx]

**Additional file 3: Table S1**. Proportion of redundant peptides (multiple peptides of a same sequence) in decoy databases for simulated proteogenomic search.

| Database (target + decoy) | 1T1D_y_ + 2D_y_  or 1T1D_h_ + 2D_h_ | 1T2D_y_ + 3D_y_  or 1T2D_h_ + 3D_h_ | 1T5D_y_ + 6D_y_  or 1T5D_h_ + 6D_h_ |
| --- | --- | --- | --- |
| Yeast | 0.29% | 0.43% | 0.74% |
| Human | 0.85% | 1.08% | 1.50% |
