# Supplementary material for: Evaluating the effect of database inflation in proteogenomic search on sensitive and reliable peptide identification
Source: BMC Genomics. 2016 Dec 22;17(Suppl 13):1031. doi: 10.1186/s12864-016-3327-5 (PMC5259817; doi:10.1186/s12864-016-3327-5)
Supplement: Additional file 4: Figure S2. — Workflow of separate filtering FDR methods (DOCX 55 kb) [file 12864_2016_3327_MOESM4_ESM.docx]

Additional file 4: Figure S2


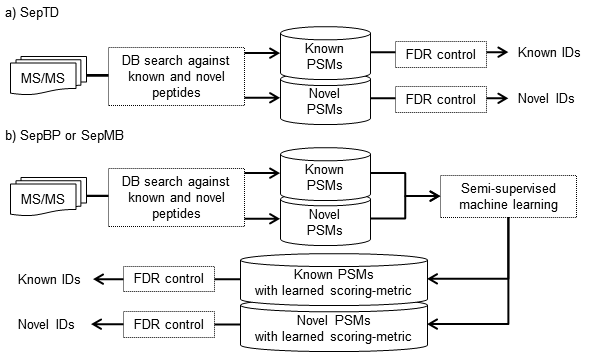


**Figure S2.** Workflow of separate filtering FDR methods. TD: target-decoy search strategy. BP: TD with a refined scoring-metric calculated by the self-boosted Percolator. MB: mixture model-based method. Here, SepTD, SepBP, and SepMB denote separate filtering of known and novel (or simulated novel) peptides with TD, BP, and MB, respectively. In SepTD, PMSs were divided into known and novel groups after database search. Then TD was applied to each PSM group separately. In SepBP or SepMB, semi-supervised machine learning techniques were applied to known and novel PSMs together after database search. Then the PSMs were divided into known and novel groups, and separately filtered by BP or MB.
