# Supplementary material for: Evaluating the effect of database inflation in proteogenomic search on sensitive and reliable peptide identification
Source: BMC Genomics. 2016 Dec 22;17(Suppl 13):1031. doi: 10.1186/s12864-016-3327-5 (PMC5259817; doi:10.1186/s12864-016-3327-5)
Supplement: Additional file 5: Figure S3. — Comparison of peptide identification results between a pair of simulated and real proteogenomic databases of similar sizes for yeast and human. (DOCX 61 kb) [file 12864_2016_3327_MOESM5_ESM.docx]

Additional file 5: Figure S3


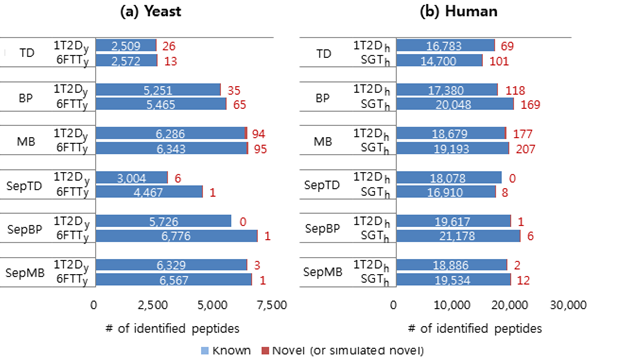


**Figure S3.** Comparison of peptide identification results between a pair of simulated and real proteogenomic databases of similar sizes for yeast (1T2D_y_ and 6FTT_y_) (a) and human (1T2D_h_ and SGT_h_) (b). Database searches were performed using Comet. The number of peptides at 1% FDR is shown. TD: target-decoy search strategy. BP: TD with a refined scoring-metric calculated by the self-boosted Percolator. MB: mixture model-based method. SepTD, SepBP, and SepMB denote separate filtering of known and novel (or simulated novel) peptides with TD, BP, and MB, respectively. The blue bars and numbers in white denote the number of known peptides. The red bars and numbers in red denote the number of novel (or simulated novel) peptides.
