# Supplementary material for: Evaluating the effect of database inflation in proteogenomic search on sensitive and reliable peptide identification
Source: BMC Genomics. 2016 Dec 22;17(Suppl 13):1031. doi: 10.1186/s12864-016-3327-5 (PMC5259817; doi:10.1186/s12864-016-3327-5)
Supplement: Additional file 6: Figure S4. — Peptide (charge 3+) identification results from search against simulated proteogenomic databases for yeast using X!Tandem. (DOCX 73 kb) [file 12864_2016_3327_MOESM6_ESM.docx]

Additional file 6: Figure S4


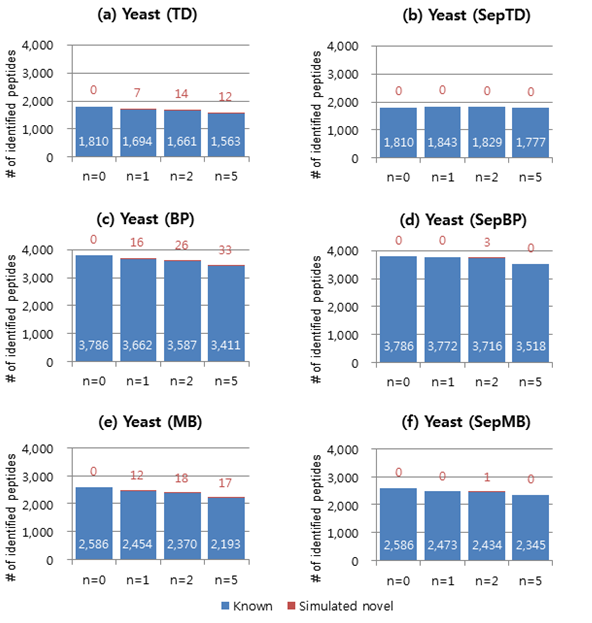


**Figure S4**. Peptide identification results from search against simulated proteogenomic databases for yeast (1T*n*D_y_) using X!Tandem (*n* = 0, 1, 2, and 5). The number of peptides with charge 3+ at 1% FDR is shown. The six search-result validation methods were used. TD: target-decoy search strategy (a). BP: TD with a refined scoring-metric calculated by the self-boosted Percolator (c). MB: mixture model-based method (e). SepTD (b), SepBP (d), and SepMB (f) denote separate filtering of known and simulated novel peptides with TD, BP, and MB, respectively. The blue bars and numbers in white denote the number of known peptides. The red bars and numbers in red denote the number of simulated novel peptides.
