# Supplementary material for: Evaluating the effect of database inflation in proteogenomic search on sensitive and reliable peptide identification
Source: BMC Genomics. 2016 Dec 22;17(Suppl 13):1031. doi: 10.1186/s12864-016-3327-5 (PMC5259817; doi:10.1186/s12864-016-3327-5)
Supplement: Additional file 12: Table S2. — Number of peptides with charge 2+ at 1% FDR identified from search against real proteogenomic databases using Comet. (DOCX 16 kb) [file 12864_2016_3327_MOESM12_ESM.docx]

**Additional file 12: Table S2.** Number of peptides with charge 2+ at 1% FDR identified from search against real proteogenomic databases using Comet. 6FTT_y_ (or 6FTT_h_): proteogenomic database constructed by 6-frame translation of yeast (or human) genome. 6FTD_y_ (or 6FTD_h_): decoy database for 6FTT_y_ (or 6FTT_h_). SGT_h_: proteogenomic database constructed by splicing information obtained from human RNA sequencing data. SGD_h_: decoy database for SGT_h_. TD: target-decoy strategy. BP: target-decoy strategy using a refined score calculated by the self-boosted Percolator. MB: mixture model-based method. SepTD, SepBP, and SepMB denote separate filtering of known and novel peptides using TD, BP, and MB, respectively.

| Database (target + decoy) | | TD | BP | MB | SepTD | SepBP | SepMB |
| --- | --- | --- | --- | --- | --- | --- | --- |
| 6FTT_y_ + 6FTD_y_ | Total | 2,501 | 3,667 | 3,567 | 3,235 | 4,159 | 3,660 |
|  | Known | 2,492 | 3,573 | 3,540 | 3,235 | 4,158 | 3,659 |
|  | Novel | 9 | 94 | 27 | 0 | 1 | 1 |
| 6FTT_h_ + 6FTD_h_ | Total | 5,695 | 5,618 | 9,099 | 10,046 | 11,216 | 10,572 |
|  | Known | 5,619 | 5,531 | 8,849 | 10,038 | 11,216 | 10,558 |
|  | Novel | 76 | 87 | 250 | 8 | 0 | 14 |
| SGT_h_ + SGD_h_ | Total | 12,143 | 15,925 | 13,865 | 12,817 | 16,747 | 14,042 |
|  | Known | 12,062 | 15,788 | 13,734 | 12,807 | 16,743 | 14,035 |
|  | Novel | 81 | 137 | 131 | 10 | 4 | 7 |
