# Supplementary material for: Evaluating the effect of database inflation in proteogenomic search on sensitive and reliable peptide identification
Source: BMC Genomics. 2016 Dec 22;17(Suppl 13):1031. doi: 10.1186/s12864-016-3327-5 (PMC5259817; doi:10.1186/s12864-016-3327-5)
Supplement: Additional file 13: Table S3. — Number of peptides with charge 3+ at 1% FDR identified from search against real proteogenomic databases using Comet. (DOCX 16 kb) [file 12864_2016_3327_MOESM13_ESM.docx]

**Additional file 13: Table S3.** Number of peptides with charge 3+ at 1% FDR identified from search against real proteogenomic databases using Comet. 6FTT_y_ (or 6FTT_h_): proteogenomic database constructed by 6-frame translation of yeast (or human) genome. 6FTD_y_ (or 6FTD_h_): decoy database for 6FTT_y_ (or 6FTT_h_). SGT_h_: proteogenomic database constructed by splicing information obtained from human RNA sequencing data. SGD_h_: decoy database for SGT_h_. TD: target-decoy strategy. BP: target-decoy strategy using a refined score calculated by the self-boosted Percolator. MB: mixture model-based method. SepTD, SepBP, and SepMB denote separate filtering of known and novel peptides using TD, BP, and MB, respectively.

| Database (target + decoy) | | TD | BP | MB | SepTD | SepBP | SepMB |
| --- | --- | --- | --- | --- | --- | --- | --- |
| 6FTT_y_ + 6FTD_y_ | Total | 1,400 | 3,001 | 2,487 | 2,216 | 3,674 | 2,521 |
|  | Known | 1,397 | 2,983 | 2,444 | 2,215 | 3,673 | 2,520 |
|  | Novel | 3 | 18 | 43 | 1 | 1 | 1 |
| 6FTT_h_ + 6FTD_h_ | Total | 2,843 | 4,462 | 3,149 | 3,875 | 5,273 | 3,467 |
|  | Known | 2,793 | 4,377 | 3,066 | 3,863 | 5,261 | 3,457 |
|  | Novel | 50 | 85 | 83 | 12 | 12 | 10 |
| SGT_h_ + SGD_h_ | Total | 5,303 | 7,470 | 5,329 | 5,586 | 7,673 | 5,291 |
|  | Known | 5,270 | 7,404 | 5,252 | 5,582 | 7,657 | 5,284 |
|  | Novel | 33 | 66 | 77 | 4 | 16 | 7 |
