# Supplementary material for: Evaluating the effect of database inflation in proteogenomic search on sensitive and reliable peptide identification
Source: BMC Genomics. 2016 Dec 22;17(Suppl 13):1031. doi: 10.1186/s12864-016-3327-5 (PMC5259817; doi:10.1186/s12864-016-3327-5)
Supplement: Additional file 14: Figure S10. — Comparison of novel peptides identified from real proteogenomic databases. (DOCX 68 kb) [file 12864_2016_3327_MOESM14_ESM.docx]

Additional file 14: Figure S10


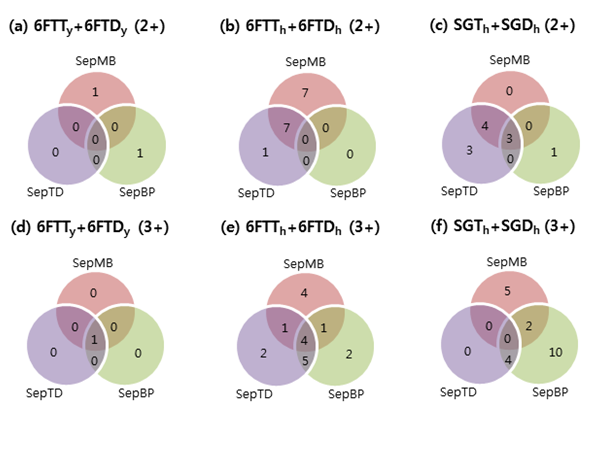


**Figure S10**. Comparison of novel peptides identified from real proteogenomic databases (‘6FTT_y_ + 6FTD_y_’ for yeast, ‘6FTT_h_ + 6FTD_h_’ and ‘SGT_h_ + SGD_h_’ for human). Database searches were performed by Comet. The number of peptides with charge 2+ ((a), (b), and (c)) and 3+ ((d), (e), and (f)) at 1% FDR is shown. Three separate filtering methods (SepTD, SepBP, and SepMB) were used for search result validation.
