# Supplementary material for: Evaluating the effect of database inflation in proteogenomic search on sensitive and reliable peptide identification
Source: BMC Genomics. 2016 Dec 22;17(Suppl 13):1031. doi: 10.1186/s12864-016-3327-5 (PMC5259817; doi:10.1186/s12864-016-3327-5)
Supplement: Additional file 15: Table S4. — Number of identified peptides at 1% FDR from the human splice graph database. (DOCX 15 kb) [file 12864_2016_3327_MOESM15_ESM.docx]

**Additional file 15: Table S4.** Number of identified peptides at 1% FDR from the human splice graph database (‘SGT_h_ + SGD_h_’). TD: the target-decoy search strategy. SepTD: TD with separate filtering of known and novel peptides. 2-stage: two-stage FDR method.

| SGT_h_ + SGD_h_ | | X!Tandem | | | Comet | | |
| --- | --- | --- | --- | --- | --- | --- | --- |
|  |  | TD | SepTD | 2-stage | TD | SepTD | 2-stage |
| Charge 2+ | Total | 8,034 | 8,957 | 11,700 | 12,143 | 12,817 | 14,731 |
|  | Known | 7,966 | 8,940 | 11,676 | 12,062 | 12,807 | 14,728 |
|  | Novel | 68 | 17 | 24 | 81 | 10 | 3 |
| Charge 3+ | Total | 3,467 | 3,840 | 5,848 | 5,303 | 5,586 | 7,358 |
|  | Known | 3,433 | 3,836 | 5,819 | 5,270 | 5,582 | 7,357 |
|  | Novel | 34 | 4 | 29 | 33 | 4 | 1 |
